# Performance on curriculum-based mathematics assessments in developmental dyscalculia: the effect of content domain and question format

**DOI:** 10.1007/s00426-024-02015-x

**Published:** 2024-08-08

**Authors:** Alison Roulstone, Kinga Morsanyi, Julia Bahnmueller

**Affiliations:** Centre for Mathematical Cognition, Department of Mathematics Education, Loughborough University, Loughborough, LE11 3TU UK

## Abstract

Developmental Dyscalculia (DD) is characterised by persistent difficulties in learning mathematical skills, which usually becomes apparent in the early years of schooling. Traditionally, DD is known to affect children’s arithmetic processing, whilst other domains of mathematics receive much less research attention. Nevertheless, contemporary diagnostic guidelines suggest that DD is linked to widespread and diverse difficulties, both within and outside of the domain of numbers. This study examined the performance (i.e., accuracy and number of questions attempted) of children on a curriculum-based mathematics assessment, considering the effect of content domains, question format (i.e., multiple-choice questions vs. constructed response questions) and test half. Participants were forty children aged 8 to 11 years old with DD (*n* = 20) and a carefully matched control group of typically developing children (*n* = 20) from primary schools in Northern Ireland. Results revealed that the DD group achieved significantly lower scores than the control group across all areas of the curriculum, and the magnitude of group differences was similar across all content domains. These findings indicate that performance in content domains other than in arithmetic may be equally informative in supporting the identification of children with DD. In addition, we found that using multiple-choice questions may support learners with DD in achieving the best outcome, and, thus, could be useful for assessing mathematics skills in dyscalculic children in classroom contexts. Nevertheless, constructed response questions may show the greatest sensitivity to identifying learners at risk, and could be the most useful in diagnostic settings.

**Supplementary Information:**

The online version contains supplementary material available at 10.1007/s00426-024-02015-x.

Developmental Dyscalculia (DD) is estimated to affect between 3 and 6% of children in primary school, yet is rarely diagnosed in the UK and most other countries (e.g., Butterworth, 2005; Kaufmann, & Von Aster, 2012; Morsanyi et al., 2018a, b). One reason for the low rates of diagnosis may be the lack of consensus around the best way to identify individuals with DD. Although using age-appropriate, standardised, curriculum-based assessments of mathematics skills is a recommended way to identify individuals with DD (cf., the Diagnostic and Statistical Manual of Mental Disorders - DSM-5; APA, 2013), it is currently unclear if all aspects of the mathematics curriculum are equally impacted, or just those relating specifically to number or arithmetic (e.g., Chinn, 2014; Holloway & Ansari, 2008; Plerou, 2014). There is also a lack of research on whether some aspects of assessments such as question format (i.e., multiple choice or constructed responses) could impact on the performance of individuals with DD, potentially influencing the results of assessments. Answering these questions could be important not just for our conceptual understanding of DD and for the purposes of diagnosis, but also in assessing mathematics skills and offering reasonable adjustments to learners with DD in educational contexts. The current study aimed to address these questions by administering a standardised, curriculum-based mathematics test to a group of children with DD and a group of typically developing (control) children, who were closely matched on several important characteristics. In addition to comparing the performance of the two groups across different content domains of the school mathematics curriculum, we also examined group differences as a function of question format and test half. In standardised, curriculum-based assessments, it is common for test items to become increasingly difficult, whilst test-takers may experience fatigue at the same time. In the following, we will first describe available evidence regarding potential effects of these factors, before presenting our own results, and discussing their implications.

Research on basic numerical skills and simple arithmetic is over-represented in the literature on dyscalculia. This includes research about subitizing (e.g., Schleifer & Landerl 2011), symbolic and non-symbolic magnitude comparison (e.g., De Smedt & Gilmore, 2011, De Smedt et al., 2013; Mussolin et al., 2010), and number line estimation (e.g., Lafay et al., 2017), with the aim of identifying the core cognitive deficits that underlie DD. By contrast, relatively few studies are focusing on more complex mathematics skills, such as word problem solving (e.g., Powell et al., 2009; Swanson, 2011) or understanding fractions (Mazzocco et al., 2013). Nevertheless, the primary focus on basic skills does not align very well with the official diagnostic criteria for DD. According to the Diagnostic and Statistical Manual of Mental Disorders (DSM-5; APA, 2013), key symptoms of DD (or Specific Learning Disorder in Mathematics) include difficulties mastering number sense, memorisation of number facts, fluency of calculation and mathematical reasoning. Additionally, the DSM-5 recommends that, when establishing a diagnosis of DD, children should be assessed on age-appropriate, standardised curriculum-based measures of mathematics skills. It is expected that individuals with DD will score at least 1.5 SD below the population mean (i.e., below the 7th percentile) on these tests. Curriculum-based mathematics assessments usually focus on all areas of the school mathematics curriculum, including shapes, data handling and measurement, in addition to arithmetic skills, computation, and mathematical reasoning. Nevertheless, it is not specified in the DSM-5 whether all these domains are expected to be equally affected. Indeed, arithmetic difficulties are often considered a hallmark of DD and several publications refer to dyscalculia as a specific disorder of arithmetic skills (e.g., Andersson & Östergren, 2012; Fortes et al., 2016; Landerl & Moll, 2010; Kaufmann & von Aster, 2012; Moll, Kunze, Neuhoff, Bruder, & Schulte-Korne, 2014). Historically, the International Classification of Diseases (10th edition, ICD-10; WHO, 1994) definition of dyscalculia also emphasized an impairment in arithmetic skills (i.e., basic computations), rather than broader mathematical skills. Nevertheless, the latest edition of the ICD (ICD-11, 2022) adopts a wider definition, also encompassing higher level mathematics skills, and mathematical reasoning.

Whereas current diagnostic recommendations by the DSM-5 (APA, 2013) and ICD-11 (WHO, 2022) acknowledge that dyscalculia affects mathematics achievement beyond arithmetic skills, there is a lack of detail regarding whether all areas of the school mathematics curriculum are expected to be equally impaired. Therefore, the main aim of the current study was to examine children’s performance in a curriculum-based assessment to determine the specific areas where dyscalculic learners may experience the greatest challenge. This question is important not only in terms of the most appropriate diagnostic approaches, but also for the development of appropriate intervention methods (i.e., so that support can be targeted at the areas of the math curriculum that are causing the greatest challenge).

Regarding the effect of question format, age-standardised, curriculum-based mathematics assessments typically utilise both multiple-choice questions (MCQs) and constructed response questions (CRQs). MCQs require an individual to select a correct answer or multiple answers from a given list of items. By contrast, CRQs are open-ended, requiring the learner to generate their own responses. Both MCQs and CRQs can be used to represent mathematics tasks with varying levels of complexity (ranging from simple to complex). Although, there has been much debate around the efficacy of using MCQs to judge the depth of children’s mathematical understanding, MCQs can provide a quick and easy way to measure performance (Dehnad et al., 2014; McKenna, 2018). Yet, evidence suggests that test scores can be positively influenced by guessing behaviour (Espinosa & Gardeazabal, 2010). Evidence also exists that gender differences in performance may arise in MCQs because boys exhibit more risk-taking behaviour (i.e., boys are more likely to guess on MCQs than girls; Beller & Gafni, 2000a; Riener & Wagner, 2017).

Whilst there are many studies that discuss gender differences in answering MCQs and CRQs (Baldiga, 2014; Beller & Gafni, 2000b; Ben-Shakhar & Sinai, 1991; Bolger & Kellaghan, 1990; Garner & Engelhard, 1999; Riener & Wagner, 2017), there is limited evidence available about how question format could affect the performance of children with DD. Powell (2012) examined the effects of response format in assessments for children with math learning disabilities. This study indicated that the use of MCQs may promote stronger performance amongst children with severe mathematical difficulty. One potential explanation was that children with math difficulties may find it easier to select a correct answer than having to construct their own response. Similarly, a study by Schulte et al. (2001) found that children with disabilities performed relatively better on MCQs than CRQs when compared to typically developing children (Schulte et al., 2001). However, it is important to note that similar findings have not been reported in other studies undertaken in secondary school settings (Bridgeman, 1992). Thus, it is currently an open question whether MCQs may give dyscalculic children the best opportunity to perform well in mathematics assessments.

It is also unclear if test items presented in an MCQ format may reduce or increase cognitive load (i.e., the amount of information held and co-ordinated in working memory to find a solution to the problem). Specifically, the way items are presented, as well as the content domain, may combine in different ways to affect children’s working memory processes. Whilst there is some evidence that MCQs may reduce test anxiety in students by presenting the correct answers (Naveh-Benjamin et al., 1981), MCQs may also involve tempting distractors, which could be particularly challenging for children with mathematical difficulties and/or impairments in attentional and inhibitory processes. Thus, to address these research gaps, we investigated whether children with DD performed relatively better on MCQs than CRQs and whether they also attempted more MCQs than CRQs, suggesting increased confidence, greater attention and motivation to engage with these kinds of questions.

A final aim of this study was to examine the effects of test half by comparing the performance of the two groups (DD vs. Control), across the first and second halves of the test by examining accuracy and the number of questions attempted (i.e., any written response to attempt to answer the question). Curriculum-based tests are often lengthy and require sustained attention and mental effort, while the difficulty of test items usually also increases towards the end of the test. We expected that these factors may lead to larger differences between the DD and control groups in the second half of the test. Reasonable adjustments for dyslexia often include the provision of rest breaks during exams, which are aimed at restoring concentration (e.g., Kindersley, 2010). Such adjustments may be recommended for dyscalculic learners as well, in case our findings suggest that their performance was disproportionately affected in the second half of the test.

## The current study

To address the questions outlined above, this study compared the performance of two groups of children on a curriculum-based mathematics assessment. Twenty children matching a cognitive profile consistent with a diagnosis of DD (DD; *n* = 20) and a carefully matched group of 20 children with age-expected mathematical skills (Control; *n* = 20) participated in the study. Performance was analysed using secondary data collected by Morsanyi et al. (2018b), using the Mathematics Assessment for Learning and Teaching (MaLT) standardised curriculum-based test (Williams et al., 2005). Performance was measured across six domains of the math curriculum: (i) Counting and understanding number; (ii) Knowing and using number facts; (iii) Calculating; (iv) Understanding shape; (v) Measuring; and (vi) Handling data. In addition to investigating performance across the six content domains, this study also investigated the effects of question format (MCQs vs. CRQs) and test half. Performance (i.e., items answered correctly) and response behaviours (i.e., items attempted) were analysed. Items attempted are defined as any effort to answer the question, including evidence of working out, drawings, calculations, or any marks on the paper indicating mathematical thinking. This study was done to develop a better understanding of the factors that may impact the performance of children with DD in standardised curriculum-based mathematics assessments.

## Method

### Participants

This study involved secondary data analyses based on data collected by Morsanyi et al. (2018b). Participants were forty children aged between 8 and 11 years old (attending Years 5, 6 and 7), recruited from seven primary schools in Northern Ireland. The study comprised of two groups. Twenty children showing persistent difficulties in learning mathematics, consistent with a diagnosis of DD, and 20 children presenting with average (age-expected) mathematical skills. All children in the two groups were recruited from the same classrooms to ensure similar educational experiences^1^. The schools represented a mixture of urban and rural schools. The children in the groups were well-matched on age (DD: *M* = 113.75 months *SD* = 8.66; Control: *M* = 117.70 months *SD* = 8.11; *p* = .145); gender (*p* = .350); eligibility to free school meals (which can be considered as a proxy for socio-economic status (DD: 75%; Control: 75%; *p* > .999); reading ability (assessed with the *Hodder Group Reading Test II;* Vincent & Crumpler, 2007; *p* = .262); and verbal and non-verbal intelligence (assessed with a short version of the *Wechsler Intelligence Scale for Children-fourth edition;* Wechsler, 2003, *p* = .940). It should be noted that children in both groups had lower than average IQ (DD: *M* = 87.05 *SD* = 9.25; Control: *M* = 86.85 *SD* = 7.32) and reading ability (DD: *M* = 86.70 *SD* = 10.19; Control: *M* = 89.70 *SD* = 5.91; *p* = .262) and were more likely to be eligible to free school meals than the general pupil population in Northern Ireland. Nevertheless, all children’s reading and IQ scores were within the normal range. The children in the DD group had persistent, severe difficulties with learning mathematics, as evidenced by the school’s records of their performance on the standardised, norm-referenced *GL Assessment Progress in Maths* (PiM) tests that they completed in previous school years. More specifically, children in the DD group had to perform at least one standard deviation below the population mean (corresponding to a standard score of 85) during all academic years where results were available. Additionally, we only included children in our sample if results were available from at least two previous academic years to ensure that the children displayed persistent difficulties. Children with any neurodevelopmental disorders (other than persistent difficulties with mathematics), or any sensory or neurological difficulties, were excluded from both groups. There were three children in the sample who were speaking English as an Additional Language (EAL). Nonetheless, all children had been educated in English for at least three school years, and were judged to have adequate English skills by the researchers. The study received ethical approval from the Faculty’s Human Ethics Committee. Informed consent was obtained from the parents of the children who participated in the study. Children also gave their verbal assent to take part. A more detailed description of the selection process can be found in Morsanyi et al. (2018b).

### Materials

Children’s mathematics performance was assessed using the *Mathematics Assessment for Learning and Teaching* (MaLT) test (Williams et al., 2005) which assesses six content domains of mathematics. Test items cover: (i) CN - Counting and understanding number - these questions typically involved counting forwards and backwards in sequences (e.g., 5, 9, _ 17, 21,25) (ii) NF - Knowing and using number facts - these questions required children to recall and apply number facts e.g., (5 × 7 - ▢  = 29) (iii) CA - Calculating - these questions involved applying arithmetic skills to solve word problems (e.g., children were asked “What number is ten less than 4000?”); (iv) SH - Understanding shape - these questions involved recognising properties of shape, symmetry, rotation and reflection (e.g. children were asked to “circle the shapes that have just one right angle.”) (v) ME - Measuring - these questions involved measuring area, perimeter, time, temperature and using units of measures, centimetres and grams (e.g. children were presented with a shape drawn on a grid of squares and asked “What is the distance round the outside of the shape in centimetres?”) and (vi) HD - Handling data – these questions involved interpreting information from tables, graphs, bar charts and pictograms (e.g., children were presented with a pictogram where they needed to identify “how many ice creams were sold on Wednesday?”). Each question was read aloud by the test administrator and the mathematics assessment was group-adminstered. Children in Year 5 completed the MaLT9 version, and children in Years 6 and 7 completed the MaLT10 test. In the current sample, the internal consistency of the tests was *α* = 0.88 for the MaLT9 and *α* = 0.92 for the MaLT10. Both MaLT assessments had a maximum score of 45 marks, although the question format, and the number of items within each curriculum content domain varied on each test version in accordance with children’s age and related mathematical development. Whereas the MaLT9 is more heavily weighted towards items about counting and understanding number, and knowing and using number facts, the MaLT10 is more heavily weighted towards calculation skills. Otherwise, the number of items within each strand (and potential marks) were relatively similar overall across both versions, as shown in Table 1.

**Table 1:** The distribution of test items according to content domain, question format and test version

Test	Content domain						Question format		Total
	CN	NF	CA	SH	ME	HD	MCQ	CRQ	
MaLT9	12	6	3	8	9	7	16	29	45
MaLT10	11	2	12	6	7	7	6	39	45

*Note* CN - Counting and understanding number; NF – Knowing and using number facts; CA – Calculating; SH – Understanding shape; ME – Measuring; HD – Handling data; MCQ – Multiple-Choice Questions; CRQ – Constructed Response Questions

### Procedure

#### Administration of the MaLT test

Participants completed a range of tasks during three testing sessions in the original study (Morsanyi et al., 2018b). However, because the current study focuses solely on a comparison of mathematical performance between the DD and a typically developing control group, the description of the procedure is restricted to the administration of the MaLT test only. In accordance with their level of education, the MaLT9 test was administered to 27 children (DD = 14, Control = 13), and the MaLT10 test was administered to 13 children (DD = 6, Control = 7). There were no practice questions included in either of the tests. Test administrators read each question out loud to the children, and the test was group administered. The children were given time to respond to each question. Although the children were not familiar with the MaLT test layout, they had had experience of completing a similar curriculum-based mathematics test (i.e., the Progress in Maths test - see https://gl-assessment.co.uk) prior to undertaking the MaLT test. Children were given identical verbal and written instructional reminders at the beginning of the assessment. The assessment was conducted in a familiar classroom setting. Children were allowed a maximum of 45 min to complete the test and the test was group-administered.

#### Analysis of MaLT test scores

The children’s written responses in the MaLT test papers were individually coded by item. In line with the marking policy, a correct response was scored as one (1 = correct response) and incorrect scored zero (0 = incorrect response). Total scores and percentage of correct responses were calculated for each of the six curriculum areas. After this, mean percentage scores were calculated for the proportion of items answered correctly for each category of response, so comparisons could be made between the two groups. Finally, the percentages of items attempted by content domain, question format (MCQs vs. CRQs) and test half (1st Test Half vs. 2nd Test Half) were also calculated for both groups. The data that support the findings of this study are available at:

- https://osf.io/q8ums/?view_only=18937e6b5448436cb4b888f17c5a5df7.

## Results

Descriptive statistics for overall performance scores are reported by curriculum content domain (counting and understanding number, knowing and using number facts, calculating, understanding shape, measuring and data handling), question format (MCQs vs. CRQS) and test half (1st Half vs. 2nd Half) can be found in Appendix A. All analyses were first run taking into account test version (i.e., MaLT9 or MaLT10), but because it did not change any of the main results, we report the simpler analyses below.

### Group differences in overall mathematics performance

There was a large discrepancy in overall performance on the MaLT between the DD and Control group (*t*(38) = 8.26, *p* < .001, Hedges *g* = 2.56)^2^, with the DD group achieving significantly lower total scores (*M* = 8.60, *SD* = 3.89) than the Control group (*M* = 21.80, *SD* = 6.00).

### Effects of content domain on group differences

**Fig. 1:**
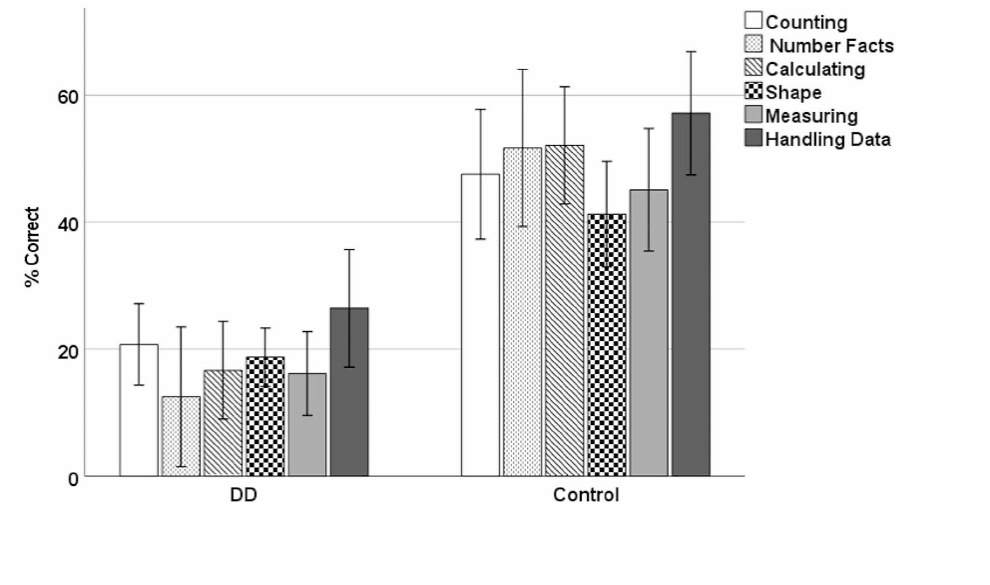
Percentage of correctly answered items across content domains by groups (error bars represent 95% confidence intervals)

First, we ran a 6 × 2 mixed ANOVA with the six curriculum content domains (Counting and understanding number vs. Knowing and using number facts vs. Calculating vs. Understanding shape vs. Measuring vs. Handling data) as within-subject factor, and group (DD vs. Control) as between-subject factor. The percentage of correctly answered items within each content domain was used as the dependent measure (see Appendix A - Supplementary Table 1). There was a significant main effect of content domain (*F*(5,190) = 2.80, *p* = .018, $\:{\eta\:}_{p}^{2}$ = 0.07). Bonferroni-corrected post-hoc *t* tests indicated a significant difference in performance between understanding shape and handling data (*p* = .029). The main effect of group was also significant (*F*(1,38) = 68.05, *p* < .001, $\:{\eta\:}_{p}^{2}$ = 0.64). However, the interaction between content domain and group was not significant (*p* = .269; see Fig. 1). This suggested that although the relative difficulty of questions across content domains varied, the magnitude of group differences was similar across content domains.

### Effects of question format and test half on group differences

**Fig. 2:**
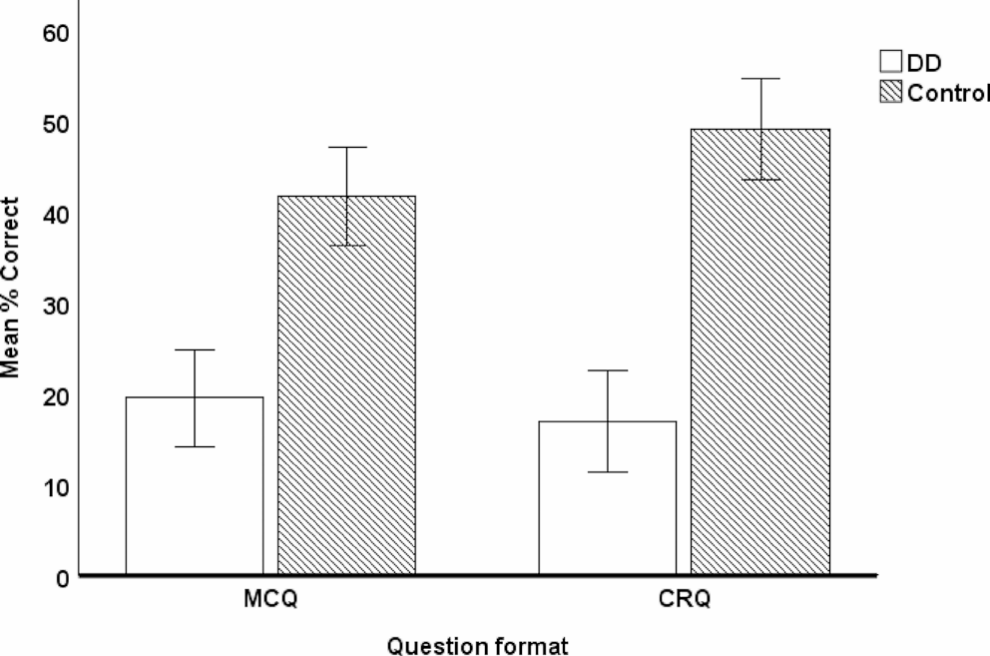
Percentage of correct responses split by question format and group (error bars represent 95% confidence intervals)

We also investigated the effect of question format and test half on group differences in the percentage of correctly answered questions. In particular, we ran a 2 × 2 × 2 mixed ANOVA with question format (MCQ vs. CRQ) and test half (1st half vs. 2nd half) as within-subject factors, and group (DD vs. Control) as between-subject factor on the percentage of correctly answered questions. There was a significant main effect of group (*F(*1,38) = 77.42, *p* < .001, $\:{\eta\:}_{p}^{2}$ = 0.67) with more items answered correctly by children in the Control group than in the DD group, as shown in Appendix A - Supplementary Table 2. Additionally, there was a significant interaction between question format and group (*F*(1,38) = 4.73, *p* = .036, $\:{\eta\:}_{p}^{2}$ = 0.11) with larger performance differences seen between groups on CRQs than MCQs (Fig. 2).

In addition, there was a significant main effect of test half (*F*(1,38) = 4.29, *p* = .045, $\:{\eta\:}_{p}^{2}$ = 0.10) with a greater number of items answered correctly in the first half as compared to the second half of the test. Otherwise, there were no other significant main effects or interactions.

**Fig. 3:**
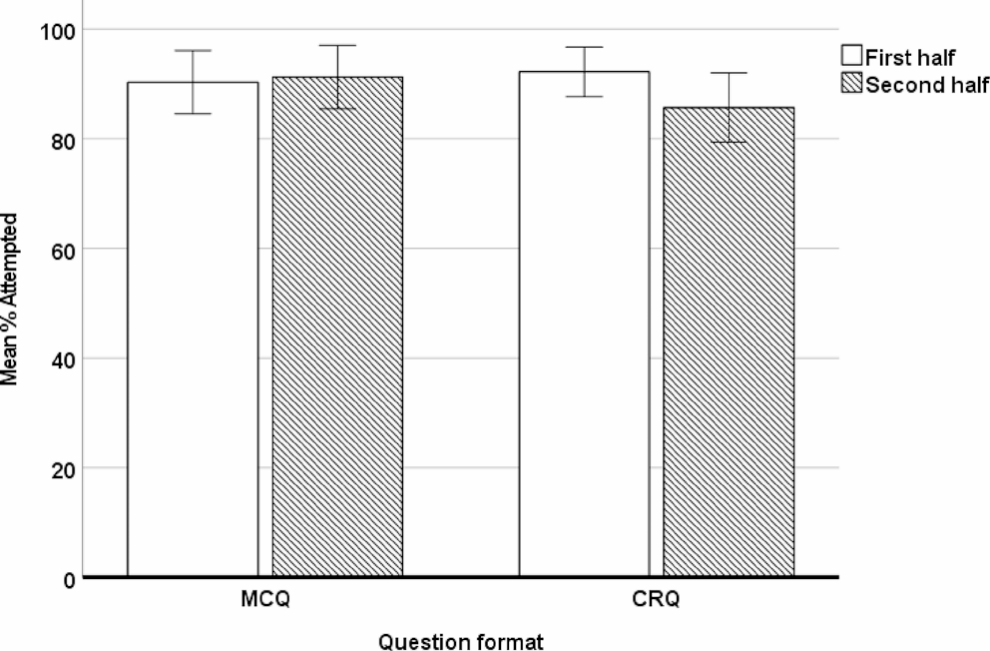
Percentage of attempted questions split by question format and test half (error bars represent 95% confidence intervals)

Next, we examined the effects of question format and test half on group differences in the proportion of items attempted. We ran a 2 × 2 × 2 mixed ANOVA with question format (MCQ vs. CRQ) and test half (1st half vs. 2nd half) as within-subject factors, and group (DD vs. Control) as between-subject factor. The percentage of MCQs and CRQs attempted was used as the dependent measure. There was a main effect of group (*F*(1,38) = 11.18, *p* = .002, $\:{\eta\:}_{p}^{2}$ = 0.23), with a higher percentage of items attempted by the control group compared to the DD group, as shown in Appendix A - Supplementary Table 3. There was also a significant interaction between question format and test half (*F*(1,38) = 12.57, *p* = .001, $\:{\eta\:}_{p}^{2}$ = 0.25) indicating that the difference in attempted questions between the first and second half was bigger for CRQs than MCQs. There were no other significant main effects or interactions (see Fig. 3).

## Discussion

The main aim of this study was to test differences between the performance of children with DD and matched controls on a standardised, curriculum-based mathematics assessment. Our study offered a detailed analysis of children’s performance, and the number of items attempted by the children, across content domains, question formats and test half. Our results indicated that children in the DD group experienced significant and comparable challenges in answering questions across all areas of the mathematics curriculum, as compared to their peers. Thus, although arithmetic difficulties have long been considered a hallmark of dyscalculia (e.g., Lewis et al., 1994; Moll et al., 2014), children in the DD group did not show more pronounced difficulties in this area than in other content domains. In fact, they showed similar levels of difficulties to arithmetic in understanding shape, a content area that did not require the processing of symbolic number representations. These findings are important because they support the notion that dyscalculia extends to a broader set of mathematics skills, which is now also reflected in the official diagnostic criteria for specific learning disorder in mathematics in the DSM-5 (APA, 2013), as well as in the ICD-11 (WHO, 2022). It has already been demonstrated that DD is characterised by impairments that extend beyond the domain of mathematics, such as problems with verbal and visuo-spatial short-term memory and working memory (e.g., Attout & Majerus, 2015; Bull & Scerif, 2001; Bull et al., 2008; Geary, 2004; Hitch & Mcauley, 1991; Mammarella et al., 2015; McLean & Hitch, 1999; Passolunghi & Siegel, 2001; Rotzer et al., 2009), inhibitory control (Blair et al., 2008; Clayton & Gilmore, 2015; Gilmore et al., 2013; Pickering, 2001; Szucs et al., 2013), order processing (Morsanyi et al., 2018a, b), visual perception (e.g., the ability to rotate and manipulate images mentally; Cheng et al., 2018; Neuburger et al., 2011), attentional function (Ashkenazi et al., 2009; Swanson, 2011) and logical reasoning (Morsanyi et al., 2013). These domain-general deficits may contribute to lower mathematical performance across a variety of content domains and task formats (Peters & Ansari, 2019).

Although the magnitude of group differences did not depend on content areas, our findings suggest that there are other factors that may influence dyscalculic children’s test performance relative to their typically developing peers. Specifically, we found that dyscalculic children showed relatively better performance on MCQs than CRQs, as compared to the control group. This is in line with other studies that showed that children with disabilities (including children with severe mathematics difficulties) performed relatively better on MCQs than other question types (Powell, 2012; Schulte et al., 2001). This finding is important, as it suggests that, when assessing children’s knowledge in school settings, MCQs may help children with mathematics difficulties to show their full potential. Learners typically prefer MCQs because they perceive them to be easier than other formats, since they are given the correct answer, instead of having to construct their own response (Butler, 2018). Indeed, all children were more likely to attempt MCQs than CRQs in the second half of the test, where item difficulty was higher. Whereas MCQs can be helpful to offer in school-based assessments, CRQs are more sensitive to group differences, and, for this reason, they may be more useful for diagnostic purposes than MCQs.

Performance of all children declined in the second half of the assessment. This is unsurprising, given that the items got increasingly difficult as children progressed through the test, as also documented in the test manual. As an additional factor, the children may have experienced cognitive fatigue over the course of the test, which lasted 45 min. Whereas it is not possible to disentangle the effects of item difficulty and fatigue in our study, what our results show is that dyscalculic and non-dyscalculic children’s performance declined to a similar degree in the second half of the test. In other words, in contrast with our expectation, DD children were not affected disproprortionately by item difficulty and any potential effects of cognitive fatigue. It could be tempting to interpret this finding as an indication that DD children would not benefit from a provision of rest breaks when attempting longer tests in classroom settings. However, the lack of effect might be the consequence of comparing their performance to a control group who were closely matched to the DD group on their cognitive abilities. In typical classroom settings, DD children are likely to be charecterised by some cognitive impairments, as compared to their classmates without DD, and rest breaks may still be advantageous.

Children needed to listen, and respond to each question as they were read aloud by the administrator of the test. An analysis of omitted questions showed that all children attempted the final questions of the test, suggesting that they kept up with the group during test administration. Nevertheless, children with DD attempted fewer questions throughout the test (including both MCQs and CRQs). Indeed, whereas children in the control group attempted almost all questions (96%), the average proportion of attempted questions was substantially lower in the DD group (81%). This points at the possibility that children with DD skipped a few questions in order to be able to keep up with the pace of test administration. Allowing more time for dyscalculic children to complete test items (as it is common practice in relation to other learning difficulties, such as dyslexia – e.g., Kindersley, 2010) and/or encouraging them to at least give each question a try could result in better performance. Indeed, children in the DD group may have required more time to interpret questions. Thus, higher omission rates may have been the consequence of children in the DD group still attending to the previous question whilst the next question was introduced and, therefore, needing to skip the question to keep up with the group as questions were read out loud. Thus, it may be advantageous for dyscalculic learners to be allowed longer time during curriculum-based assessments, and they may need more explicit encouragement to try. This may be best implemented in a one-to-one or small group setting, so that the pace of test administration can be adjusted to meet their needs.

## Strengths, limitations, and future directions

Although using standardised achievement measures is a recommended, and widely used method to identify children at risk of mathematical difficulties, to the best of our knowledge, this was the first study to provide a detailed analysis of group differences in performance (examining the effects of content domains, question format and test length) in a curriculum-based, standardised test, between children with DD and their typically developing peers. Examining all these factors and their interactions together was a particular strength of our study. The samples were also carefully matched on various cognitive and contextual variables (including their age, gender, IQ and reading skills, socio-economic status, and whether they spoke English as their first language), and children with any neurodevelopmental disorders (other than persistent difficulties with mathematics), or any sensory or neurological difficulties, were excluded from both groups. Children in the DD group also displayed persistent and severe difficulties with mathematics over more than two school years. Despite these rigorous selection criteria, we did not have enough information about the children in the DD group to be able to establish a dyscalculia diagnosis, as this would have required individual assessment by an experienced clinician. Additionally, our sample was restricted to forty children aged between 8 and 11 years old, and children in both groups had slightly lower than average IQ and reading abilities, as well as coming from lower socio-economic backgrounds than the general population. This is partially a reflection of the typical demographic and cognitive characteristics of children with dyscalculia, who, on average, are characterised by lower IQ and reading scores, and are more likely to come from lower socio-economic status backgrounds than children without dyscalculia (e.g., Emerson, 2012; Morsanyi et al., 2018a, b). Nevertheless, it would be advantageous to extend this work in the future to larger populations and children from more diverse socio-ecomomic backgrounds.

In accordance with their age and level of education, the children in the sample completed one of two different versions of the MaLT. These versions differed in the proportion of MCQs vs.CRQs, and in the distribution of items across content domains. For this reason, we converted our data into proportion of correct responses, rather than working with raw scores. Nevertheless, there was no evidence to indicate that test version affected any of our results regarding group differences (i.e., our findings appeared to be robust across the two different MaLT tests).

In terms of future directions, in addition to conducting similar studies with larger samples and different age groups, it would be important to investigate further the differences in performance between dyscalculic and non-dyscalculic individuals on MCQs and CRQs. In the current study, it has been found that dyscalculic children performed relatively better on MCQs than on CRQs, as compared to the control group. Nevertheless, MCQs typically involve salient distractors, and hypersensitivity to interference (e.g., De Visscher & Noël, 2013) and inhibition difficulties (e.g., Szucs et al., 2013) are commonly reported in dyscalculia. Thus, it would be important to further investigate the boundary conditions of when MCQs offer an advantage in assessment situations to children with dyscalculia (Williams et al., 2007). Specifically, in relation to distractor items, the types of distractors that most strongly affect the performance of children with DD may differ from the distractors that non-dyscalculic children are the most sensitive to. This question could be investigated by analysing incorrect response patterns on MCQ tests or by recording eye-gaze patterns while children are working through the tasks (see e.g., Lindner et al., 2014).

Another important future direction would be to investigate the effects of reasonable adjustments (such as extra time and one-to-one or small group administration of tests) on dyscalculic children’s performance (Lovett, 2010; Woods & Reason, 1999). A final point of consideration is that our study suggests that dyscalculic children experience similar levels of difficulties across different domains of the curriculum. However, we did not analyse children’s performance in detail within different curriculum domains. Thus, it would be important to conduct studies that investigate performance in the domains of mathematics that have not received much research attention so far, including understanding shape, measurement, and handling data.

## Conclusion

Our findings have some important implications regarding our understanding of the nature of mathematics difficulties in dyscalculia, as well as some practical implications, in relation to identifying children at risk of dyscalculia, and for administering curriculum-based standardised mathematics assessments in the classroom to dyscalculic learners. Notably, we found that significant performance differences existed in all content domains of the mathematics curriculum between children in the two groups. Thus, our findings indicate that dyscalculic learners may experience comparable challenges in all areas of the mathematics curriculum, and not just those specifically related to arithmetic or numbers. This suggests that low mathematics performance in any content domain may be diagnostically informative. Therefore, although no single data source is sufficient for a clinical diagnosis of DD, persistent low scores on age-standardised mathematics assessments may provide a valuable source of evidence to support a referral for clinical assessment of DD, regardless of the exact content of these tests. Additionally, group differences were more prominent in the case of CRQs, which suggests that these questions could be particularly informative in diagnostic contexts. Our findings also point at the potential usefulness of offering some adjustments to children with dyscalculia to support their performance in school-based assessments, such as allowing additional time, encouraging them to attempt all questions, and administering mathematics tests in one-to-one or small group settings. Finally, MCQs may give children with DD the best opportunity to show their full potential.

## Electronic supplementary material

Below is the link to the electronic supplementary material.

Supplementary Material 1

## Data Availability

The data that support the findings of this study are available on OSF at: https://osf.io/q8ums/?view_only=18937e6b5448436cb4b888f17c5a5df7.

## Footnotes

1 Children in Northern Ireland start primary school after their 4th birthday, at a younger age than in most other countries. Children in the school years 5, 6 and 7 are between 8 and 11 years of age.

2 Due to small sample sizes, we report Hedges’ *g* rather than Cohen’s *d* effect size statistics. However, the interpretation of these results is the same, with an effect size of 0.2 considered small, 0.5 is medium, and 0.8 is large.
