# Supplementary material for: Performance on curriculum-based mathematics assessments in developmental dyscalculia: the effect of content domain and question format
Source: Psychol Res. 2024 Aug 8;88(8):2444–54. doi: 10.1007/s00426-024-02015-x (PMC11522108; doi:10.1007/s00426-024-02015-x)
Supplement: Supplementary file 1 — Supplementary Material 1 [file 426_2024_2015_MOESM1_ESM.docx]

**Appendix A**

**Supplementary Table 1** *The effect of content domain on performance in the DD and Control group (Mean % of correct items)*

|  | DD | | Control | |
| --- | --- | --- | --- | --- |
| Content Domain | *M* (*SD*) | Range  Min-Max | *M* (*SD*) | Range  Min-Max |
| Counting | 20.76 (*13.72*) | 0 – 54.55 | 47.54 (*21.86*) | 8.33 – 100.00 |
| Number Facts | 12.50 (*23.49*) | 0 – 100.00 | 51.67 (*27.44*) | 0 – 100.00 |
| Calculating | 16.67 (*16.45*) | 0 – 41.67 | 52.08 (*19.66*) | 0 – 75.00 |
| Shape | 18.75 (*9.79*) | 0 – 37.50 | 41.25 (*17.78*) | 12.50 – 66.67 |
| Measuring | 16.19 (*14.09*) | 0 – 44.44 | 45.07 (*20.59*) | 22.22 – 100.00 |
| Handling Data | 26.43 (*19.81*) | 0 – 71.43 | 57.14 (*20.73*) | 28.57 – 100.00 |

*Note:* N = 40 (*n* = 20 for each group). 95% Confidence Interval.

**Supplementary Table 2** *The effect of question format and test half on the percentage of items correct in the DD and Control group*

|  | DD | | Control | |
| --- | --- | --- | --- | --- |
| Question Format & Test Half | *M* *(SD)* | Range  Min-Max | *M* *(SD)* | Range  Min-Max |
| MCQ 1^st^ Half correct | 20.63 *(17.81)* | 0 - 50.00 | 40.00 *(31.31)* | 0 – 100.00 |
| MCQ 2^nd^ Half correct | 18.38 *(12.23)* | 0 - 40.00 | 43.38 *(24.58)* | 12.50 - 100.00 |
| CRQ 1^st^ Half correct | 23.21 *(13.42)* | 0 - 47.62 | 55.24 *(14.95)* | 21.43 - 95.24 |
| CRQ 2^nd^ Half correct | 10.67 *(11.92)* | 0 – 38.89 | 42.89 *(15.69)* | 11.11 – 72.22 |

*Note:* N = 40 (*n* = 20 for each group). 95% Confidence Interval. MCQ = Multiple-Choice Questions % of items correct; CRQ = Constructed Response Question % of items correct.

**Supplementary Table 3** *The effect of question format and test half on the percentage of items attempted by children in the DD and Control group.*

|  | DD | | Control | |
| --- | --- | --- | --- | --- |
|  | *M* *(SD)* | Range  Min-Max | *M* *(SD)* | Range  Min-Max |
| MCQ 1^st^ Half attempted | 83.75 *(24.37)* | 25.00 -100.00 | 96.88 *(7.98)* | 75.00 - 100.00 |
| MCQ 2^nd^ Half attempted | 83.13 *(25.42)* | 12.50 -100.00 | 99.38 *(2.79)* | 87.50 - 100.00 |
| CRQ 1^st^ Half attempted | 86.07 *(19.69)* | 35.71 -100.00 | 98.33 *(3.00)* | 92.86 - 100.00 |
| CRQ 2^nd^ Half attempted | 76.33 *(26.86)* | 6.67 - 100.00 | 95.06 *(7.05)* | 80.00 - 100.00 |

*Note:* N = 40 (*n* = 20 for each group). 95% Confidence Interval. MCQ = Multiple-Choice Questions % of items attempted; CRQ = Constructed Response Questions % of items attempted.
